# Expression of FRS2 in atypical lipomatous tumor/well-differentiated liposarcoma and dedifferentiated liposarcoma: an immunohistochemical analysis of 182 cases with genetic data

**DOI:** 10.1186/s13000-021-01161-9

**Published:** 2021-10-25

**Authors:** Wenyi Jing, Ting Lan, Yan Qiu, Ran Peng, Yang Lu, Huijiao Chen, Min Chen, Xin He, Chen Chen, Hongying Zhang

**Affiliations:** 1 Department of Pathology, West China Hospital, Sichuan University, Guoxuexiang 37, Chengdu, 610041 Sichuan China; 2 Department of Pathology, Sichuan Cancer Hospital & Institute, Sichuan Cancer Center, Cancer Hospital affiliate to School of Medicine, University of Electronic Science and Technology of China, Chengdu, China

**Keywords:** Atypical lipomatous tumor, Well-differentiated liposarcoma, Dedifferentiated liposarcoma, Immunohistochemistry, FRS2

## Abstract

**Background:**

The fibroblast growth factor receptor substrate 2 (*FRS2*) gene is located close to *MDM2* and *CDK4* within the 12q13-15 chromosomal region. *FRS2* gene was recently found to be consistently amplified in atypical lipomatous tumor (ALT)/well-differentiated liposarcoma (WDL) and dedifferentiated liposarcoma (DDL), suggesting the detection of *FRS2* amplification could be a diagnostic tool for ALT/WDL/DDLs. However, the expression of FRS2 protein and diagnostic value of FRS2 immunohistochemistry (IHC) has not been evaluated in a large cohort of ALT/WDL/DDLs.

**Methods:**

A SNOMED search of hospital surgical pathology files from January 2007 to July 2020 identified 182 ALT/WDL/DDLs with available materials. *FRS2* fluorescence in situ hybridization (FISH) and IHC were performed on 182 ALT/WDL/DDLs and 64 control samples. The expression of FRS2 was also compared with that of classic immunomarkers (MDM2 and CDK4) of this tumor entity.

**Results:**

This study included 91 ALT/WDLs and 91 DDLs. The FISH results showed 172 of 182 (94.5%) cases were *FRS2*-amplified, and 10 cases were *FRS2*-nonamplified. Immunostaining results showed 171 (94.0%) ALT/WDL/DDLs were positive for FRS2 and 11 cases (6.0%) were FRS2-immunonegative. In 172 *FRS2*-amplified cases, 166 (96.5%) were FRS2-immunopositive, and 6 (3.5%) were negative. Among 10 *FRS2*-nonamplified ALT/WDL/DDL cases, 5 cases were FRS2-immunonegative, and 5 tumors displayed 1+ staining for this marker. In 64 control cases, none of them exhibited *FRS2* amplification. Forty-seven (73.5%) control cases were negative for FRS2 immunostaining, while 17 cases (26.5%) were FRS2-immunopositive. Fifteen of these false positive samples (15/17, 88.2%) showed 1+ positivity and only 2 cases (2/17, 11.8%) displayed 2+ positivity. In ALT/WDL/DDLs, the sensitivity of FRS2 immunostaining was slightly lower than MDM2 (FRS2 vs. MDM2: 94.0% vs 100.0%) and CDK4 (FRS2 vs. CDK4: 94.0% vs 97.0%). However, the specificity of FRS2 (73.5%) was slightly higher than that of MDM2 (67.8%) and CDK4 (64.4%).

**Conclusion:**

This study indicated that FRS2 IHC had relatively good consistency with *FRS2* FISH, suggesting that FRS2 immunostaining could be utilized as an additional screening tool for the diagnosis of ALT/WDL/DDL. It must be emphasized that *MDM2/CDK4/FRS2* especially *MDM2* FISH remains the gold standard and the most recommended method to diagnose this entity.

## Background

Liposarcoma is one of the most common soft tissue sarcomas in adults and consists of four subtypes: atypical lipomatous tumor (ALT)/well-differentiated liposarcoma (WDL)/dedifferentiated liposarcoma (DDL), myxoid liposarcoma, pleomorphic liposarcoma, and myxoid pleomorphic liposarcoma [1–3]. The ALT/WDL/DDL subgroup accounts for the majority of liposarcomas, exhibiting amplification of the chromosome 12q13-15 region, leading to the amplification of *MDM2*, *CDK4*, *HMGA2* and *CPM* [4–6]. Hence, fluorescence in situ hybridization (FISH) for the identification of *MDM2*/*CDK4* amplification or immunohistochemistry (IHC) for the detection of their overexpression were recommended as useful ancillary tools for the diagnosis of ALT/WDL/DDL [7–13].

The fibroblast growth factor receptor substrate 2 (*FRS2*) gene is also located in the 12q13-15 region close to the *MDM2* and *CDK4* genes. In 2011, Wang et al. reported the consistent amplification of the *FRS2* gene in ALT/WDL/DDLs (57 of 57, 100%) [14]. Later, Zhang et al. verified *FRS2* amplification in 15 DDLs, with an amplification frequency of 93% (14/15) [15]. Our recent work also identified that the *FRS2* gene is highly amplified in 146 ALT/WDL/DDL cases (136/146, 93.2%) [16]. These results indicated that FRS2 have diagnostic utility for this tumor group. Because the lack of commercial *FRS2* FISH probes and the evaluation of FISH requires specific equipment and experienced cytogeneticists. Furthermore, IHC is a more economical alternative and could be carried out in most hospitals in clinical practice. Hence, it is necessary to examine the utility of FRS2 IHC for the diagnosis of ALT/WDL/DDL. Moreover, FRS2 acts as a key adaptor protein in the fibroblast growth factor receptor (FGFR) pathway, subsequently activating downstream signaling pathways [17]. Hence, the detection of FRS2 protein expression in ALT/WDL/DDL may also provide clues for their therapy in the future. However, the expression of FRS2 in ALT/WDL/DDL and the utility of FRS2 IHC for the differential diagnosis of this disease spectrum have not been widely investigated. FRS2 IHC was only performed by Zhang et al. in 11 DDLs, among which 9 (82%) cases were positive for FRS2 [15].

Here, we performed FRS2 immunostaining in 182 ALT/WDL/DDL and 64 control cases, and the results were compared with *FRS2* FISH data of the 182 cases to evaluate the sensitivity, specificity and diagnostic value of FRS2 IHC for this entity. Moreover, the MDM2 and CDK4 IHC results of most cases were reviewed to compare FRS2 and these classic immunomarkers for ALT/WDL/DDL.

## Methods

### Case selection

This study was approved by the West China Hospital Institutional Review Board. A SNOMED search of the hospital surgical pathology files from January 2007 to July 2020 identified 182 ALT/WDL/DDLs with available materials for further study. Histological sections as well as previous MDM2 and CDK4 IHC slides of the tumors were reviewed independently by two pathologists with soft tissue tumor pathology expertise (H.Z. and H.C.) and 2 general surgical pathologists (W.J. and T.L.) independently. Classification was performed according to the World Health Organization criteria, and grading was carried out following the ‘modified’ Federation Nationale des Centres de Lutte Contre le Cancer (FNCLCC) grading system [2, 18]. Clinicopathological information was collected from clinical records and pathology reports. The control cases were obtained independently from clinical cases, including conventional lipoma (*n* = 25), spindle cell lipoma (*n* = 7), atypical spindle cell lipomatous tumor (*n* = 3), myxoid liposarcoma (*n =* 2), pleomorphic liposarcoma (*n* = 8), myxofibrosarcoma (*n* = 5), leiomyosarcoma (*n =* 5), schwannoma (*n* = 1), undifferentiated pleomorphic sarcoma (*n* = 3), osteosarcoma (*n =* 1), low-grade myofibroblastic sarcoma (*n =* 1), and normal fat (*n =* 3).

### Fluorescence in situ hybridization (FISH)

*FRS2* FISH was performed on 182 ALT/WDL/DDLs, among which 146 cases had archival *FRS2* FISH records that were published previously [16], and additional *FRS2* FISH analyses were performed on the remaining 36 cases. *MDM2* FISH was performed on all *FRS2* nonamplified ALT/WDL/DDL cases. FISH assays were carried out according to an established laboratory protocol [8, 19]. A bacterial artificial chromosome (BAC) clone for *FRS2* (RP11-956E11) was purchased from the Children’s Hospital Oakland Research Institute (CHORI, Oakland, CA, USA). Preparation and validation of the *FRS2* probe was performed according to a previously published study [14]. A Vysis LSI *MDM2* dual-color probe (Abbott Molecular, Des Plaines, IL, USA) was used for the *MDM2* FISH assay. Tumors were scored by two investigators counting 100 nuclei in a blinded fashion. The hemorrhage and necrotic areas were excluded as much as possible when making the evaluation. The amplification of *FRS2* or *MDM2* was defined as *FRS2*/CEP12 or *MDM2*/CEP12 ≥ 2.0, while a ratio < 2.0 was considered nonamplified and a ratio < 2.0 with more than two signals was considered polysomic for CEP12.

### Immunohistochemistry (IHC)

Immunohistochemical staining was performed on the representative sections of each case by FRS2 antibody (clone SC-8318, Santa Cruz Biotechnology, USA), MDM2 antibody (clone SMP14, ready-to-use; Dako, Carpinteria, CA) and CDK4 antibody (clone EP180, 1:100; Dako, Carpinteria, CA), using the EnVision Plus detection system (DAKO, Carpinteria CA, USA). Sections were revealed by diaminobenzidine solution (Dako, Carpintera CA, USA) and counterstained with hematoxylin. Positive and negative control sections were utilized. The results were evaluated independently by pathologists (H.Z., T.L. and Y.Q.) using the same standard, and the hemorrhage and necrotic areas were excluded as much as possible when making the evaluation. Regarding FRS2, cytoplasmic staining was considered positive, whereas nuclear staining for MDM2 and CDK4 was recorded as positive staining. IHC staining intensity was scored as none (0), weak (1), moderate (2), or strong (3), and the extent of positive tumor cells was scored as < 5% (0), 5-10% (1), 11-49% (2), 50-74% (3), or ≥ 75% (4). Cases were graded as negative – (0-1), weakly positive 1+ (2-4), moderately positive 2+ (6-8), and strongly positive 3+ (9-12) by multiplying the two scores [20].

### Statistical analysis

The sensitivity and specificity of FRS2 IHC were evaluated in the ALT/WDL and DDL subgroups to assess the ability of the immunomarker to correctly classify ALT/WDL/DDL from their histologic mimics. Student’s *t*-test was used to compare continuous variables, and McNemar and kappa tests were utilized to evaluate the agreement between *FRS2* FISH and FRS2 IHC. The chi-square test was used to compare the positive expression rates of FRS2 with the other immunohistochemical markers by SPSS version 20.0 (IBM Corp, Armonk, NY, USA). *P*-values < 0.05 were considered significant.

## Results

### Clinical and pathological findings

This study included 108 males and 74 females aged 20-83 years (median: 57 years). Tumors ranged in size from 1.2 to 45.0 cm (median: 16.0 cm). The majority of tumors were located in the retroperitoneum (*n* = 92), followed by the extremities (*n* = 36), abdominopelvic cavity (*n* = 28), trunk (*n* = 10), head and neck area (*n* = 8), scrotum/inguinal region (*n* = 6) and mediastinum (*n =* 2). The study contained 91 ALT/WDLs and 91 DDLs. The 91 ALT/WDL cases were graded into FNCLCC 1, composed of 48 lipoma-like (52.7%), 40 sclerotic (44.0%), and 3 inflammatory subtypes (3.3%). In the 91 DDLs, the dedifferentiated components had a variety of morphologic patterns. Sixty-six DDLs manifested as myxofibrosarcoma/fibrosarcoma (*n* = 53, 58.2%) and undifferentiated pleomorphic sarcoma (*n* = 13, 14.3%). Six tumors (6.6%) had low-grade fibrosarcoma-like differentiation areas. Heterologous differentiation (*n* = 11, 12.1%) and homologous pleomorphic liposarcoma-like differentiation exhibited in 8 DDLs (8.8%). Grading results showed that 39 (42.9%) DDLs were graded into FNCLCC 2, and 52 (57.1%) tumors were FNCLCC 3.

### *FRS2* FISH analysis

*FRS2* amplification was identified in 172 of 182 (94.5%) cases, including 84 ALT/WDLs (84/91, 92.3%) and 88 DDLs (88/91, 96.7%). The 10 *FRS2* non-amplified cases were *MDM2* amplified, including 7 ALT/WDLs and 3 DDLs. None of the control samples (*n* = 64) showed amplification of *FRS2*.

### FRS2 immunostaining results and the comparison with *FRS2* FISH results

#### IHC results of ALT/WDL/DDLs and control cases

**Fig. 1:**
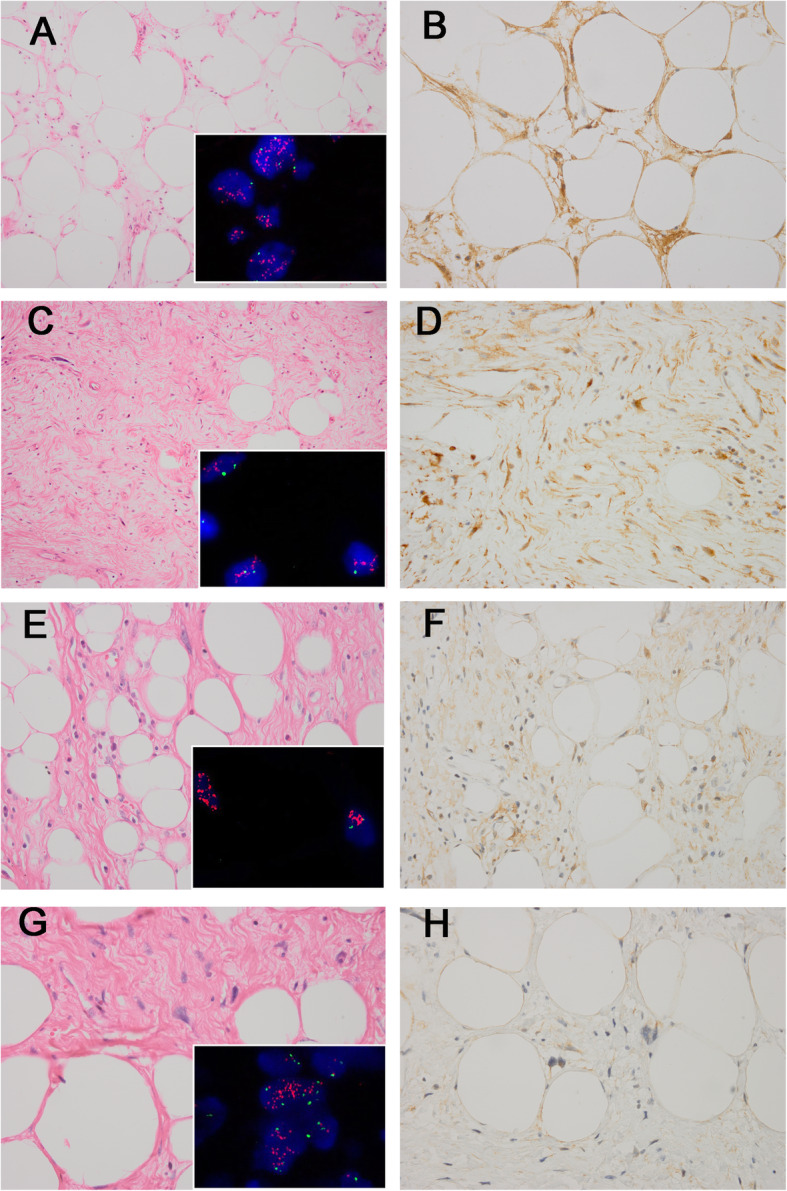
The histologic features of ALT/WDLs and corresponding *FRS2* FISH and FRS2 immunostaining results. Lipoma-like ALT/WDL(**a**) with *FRS2* amplification (**inset**), showing 3+ FRS2 expression with diffuse and strong cytoplasmic staining (**b**). Sclerotic ALT/WDL(**c**) showing *FRS2* amplification (**inset**), displayed 3+ FRS2 immunostaining, with strong FRS2 protein expression (**d**). Lipoma-like ALT/WDL(**e**) with *FRS2* amplification (**inset**), exhibited 2+ cytoplasmic staining for FRS2 (**f**). Sclerosing ALT/WDL (**g**) with *FRS2* amplification (**inset**), showing 1+ FRS2 positivity (**h**). Red signals represent *FRS2*. Green signals represent chromosome 12 centromeres. (a, c, e, g, haematoxylin and eosin; b, d, f, h, immunostaining) (original magnification, a, c × 200, b, d-h × 400)

**Fig. 2:**
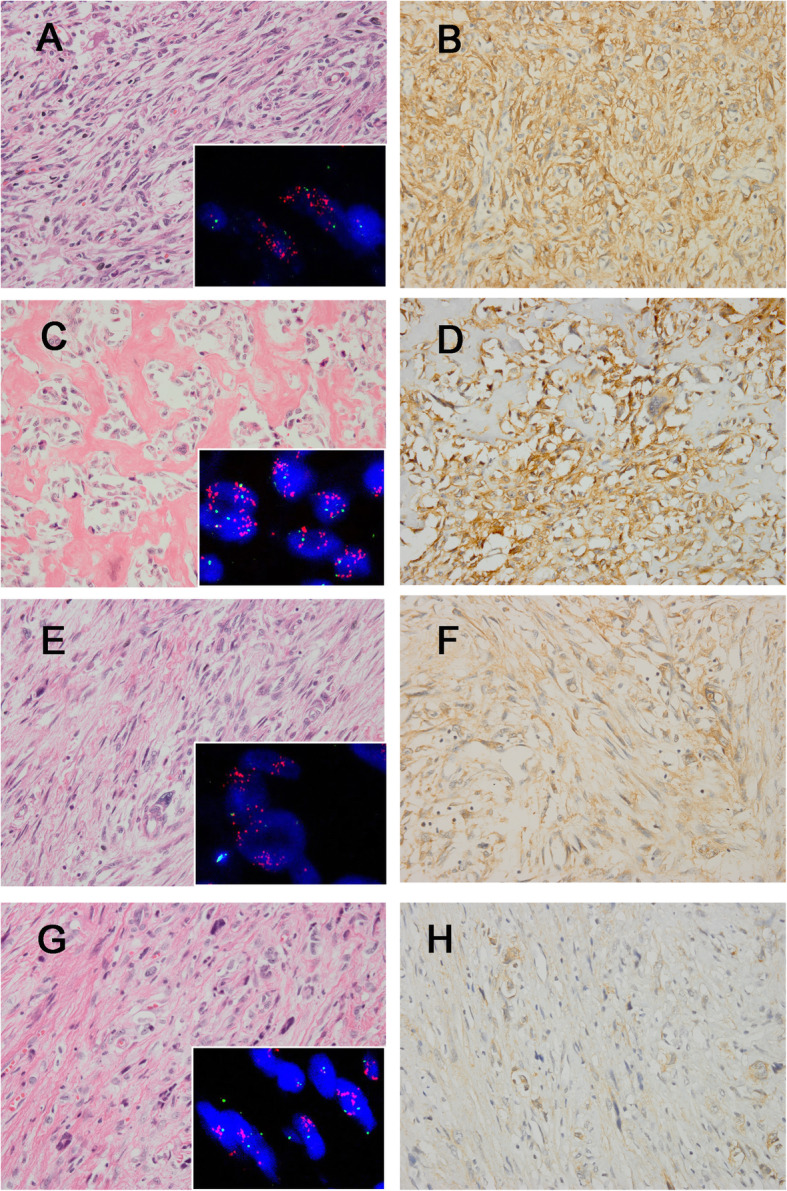
The histologic features of DDLs and corresponding *FRS2* FISH and FRS2 immunostaining results. DDL with fibrosarcoma-like differentiation(**a**), showing *FRS2* amplification (**inset**), displayed 3+ FRS2 immunostaining, exhibiting diffuse and strong cytoplasmic FRS2 expression (**b**). DDL showing osteosarcoma-like differentiation(**c**), with *FRS2* amplification (**inset**), had 3+ FRS2 staining, harboring strong FRS2 immunostaining (**d**). DDL demonstrating undifferentiated sarcoma-like differentiation(**e**), with *FRS2* amplification (**inset**), harbored 2+ FRS2 staining (**f**). DDL with leiomyosarcoma-like differentiation(**g)** harboring *FRS2* amplification (**inset**) showed 1+ positivity for FRS2 immunostaining (**h**). Red signals represent *FRS2*. Green signals represent chromosome12 centromeres. (a, c, e, g, haematoxylin and eosin; b, d, f, h, immunostaining) (original magnification a-h × 400)

The immunohistochemical results are summarized in Table 1. FRS2 cytoplasmic positivity was observed in 171/182 (94.0%) ALT/WDL/DDL cases (Figs. 1 and 2), among which 57 cases had 3+ staining (31.3%), 63 had 2+ staining (34.7%), 51 (28.0%) tumors displayed 1+ staining and 11 cases (6.0%) were FRS2-negative. In 91 ALT/WDL cases, 83 (91.2%) cases were positive for FRS2, including 50 (54.9%) ALT/WDLs with 2+/3+ staining, 33 (36.3%) cases with 1+ positivity and the remaining 8 (8.8%) cases were negative for FRS2. In terms of DDLs, 88 (96.7%) tumors were FRS2 positive, containing 18 (19.8%) cases with 1+ positivity and 70 (76.9%) tumors with 2+/3+ staining, and only 3 (3.3%) DDL cases were FRS2 negative.

**Table 1:** FRS2 immunostaining in 182 ALT/WDL/DDLs and 64 control cases

Tumor type	FRS2 immunostaining				Total
	- (%)	1+ (%)	2+ (%)	3+ (%)	
ALT/WDL/DDL	11 (6.0%)	51 (28.0%)	63 (34.7%)	57 (31.3%)	182
ALT/WDL	8 (8.8%)	33 (36.3%)	31 (34.1%)	19 (20.8%)	91
DDL	3 (3.3%)	18 (19.8%)	32 (35.2%)	38 (41.7%)	91
Control group	47 (73.5%)	15 (23.4%)	2 (3.1%)	0 (0%)	64
Normal adipose tissue	3 (100%)	0 (0%)	0 (0%)	0 (0%)	3
Benign adipose tumor					
Lipoma	20 (80.0%)	5 (20%)	0 (0%)	0 (0%)	25
Spindle cell lipoma	5 (71.4%)	2 (28.6%)	0 (0%)	0 (0%)	7
Other spindle cell tumors					
Pleomorphic liposarcoma	5 (62.5%)	2 (25.0%)	1 (12.5%)	0 (0%)	8
Myxoid liposarcoma	1 (50.0%)	1 (50.0%)	0 (0%)	0 (0%)	2
Atypical spindle cell lipomatous tumor	2 (66.7%)	1 (33.3%)	0 (0%)	0 (0%)	3
Undifferentiated pleomorphic sarcoma	2 (66.7%)	1 (33.3%)	0 (0%)	0 (0%)	3
Myxofibrosarcoma	5 (100%)	0 (0%)	0 (0%)	0 (0%)	5
Leiomyosarcoma	3 (60.0%)	2 (40.0%)	0 (0%)	0 (0%)	5
Low-grade myofibroblastic sarcoma	0 (0%)	0 (0%)	1 (100%)	0 (0%)	1
Osteosarcoma	0 (0%)	1 (100%)	0 (0%)	0 (0%)	1
Schwannoma	1 (100%)	0 (0%)	0 (0%)	0 (0%)	1

*ALT/WDL* Atypical lipomatous tumor/well-differentiated liposarcoma, *DDL* Dedifferentiated liposarcoma, − negativity, *1 +* weak positivity, *2+* moderate positivity, *3+* strong positivity

**Fig. 3:**
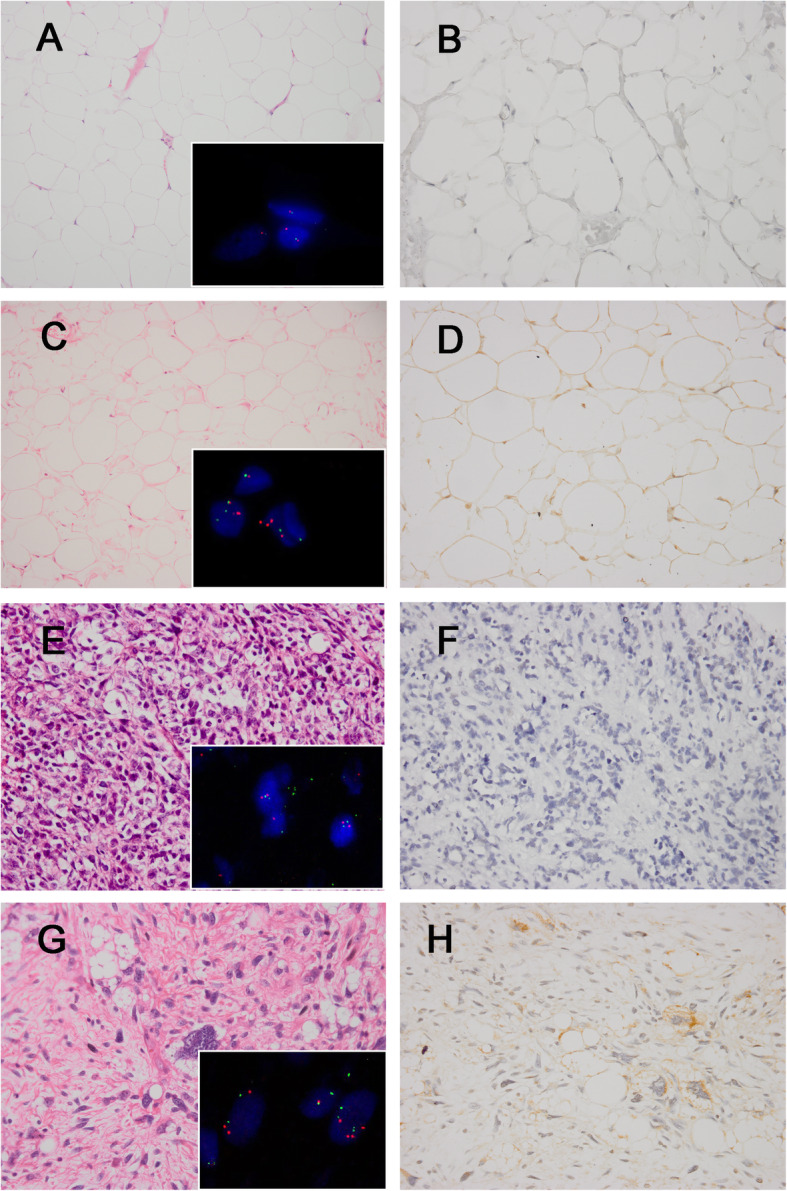
The histologic features of control tumors and corresponding *FRS2* FISH and FRS2 immunohistochemical staining results. Lipoma(**a**) without *FRS2* gene amplification (**inset**) was FRS2 immunostaining negative (**b**). Lipoma(**c**) without *FRS2* gene amplification (**inset**) but exhibited 1+ FRS2 expression (**d**). Pleomorphic liposarcoma(**e**) was *FRS2* nonamplified (**inset**) and negative for FRS2 immunostaining (**f**). Pleomorphic liposarcoma(**g**) without *FRS2* amplification showing polysomic for CEP12 (**inset**), and had 1+ FRS2 staining (**h**). Red signals represent *FRS2*. Green signals represent chromosome12 centromeres. (a, c, e, g, haematoxylin and eosin; b, d, f, h, immunostaining) (original magnification, a, c × 200, b, d-h × 400)

In 64 control samples, 47 (73.5%) cases were negative, and 17 (26.5%) cases demonstrated FRS2 positivity (Fig. 3). Fifteen of these 17 false positive cases (88.2%) displayed 1+ staining, including 5 lipomas, 2 spindle cell lipomas, 2 pleomorphic liposarcomas, 1 myxoid liposarcoma, 1 atypical spindle cell lipomatous tumor, 1 undifferentiated pleomorphic sarcoma, 2 leiomyosarcomas and 1 osteosarcoma. Only 2 cases (2/17, 11.8%) displayed 2+ staining, including 1 pleomorphic liposarcoma and 1 low-grade myofibroblastic sarcoma.

### Comparison of FRS2 IHC and FISH results in the ALT/WDL/DDL

#### Immunostaining results in *FRS2*-amplified cases

The comparison of FRS2 IHC and FISH results are summarized in Table 2. Among 172 *FRS2*-amplified cases (84 ALT/WDLs, 88 DDLs), 166 cases (96.5%) had consistent positive IHC results with their FISH results, while 6 (3.5%) *FRS2*-amplified cases were negative for FRS2 immunostaining. Statistical analysis found that FRS2 IHC had relatively good consistency with *FRS2* FISH (*P* < 0.05, kappa = 0.444). The 166 FRS2-immunopositive cases consisted of 57 cases (19 ALT/WDLs and 38 DDLs) with 3+ positivity, 63 (31ALT/WDLs and 32 DDLs) with 2+ positivity and 46 cases (31 ALT/WDLs and 15 DDLs) with 1+ staining. In the 6 FRS2-immunonegative cases, 2 cases were samples from 5 years ago, and necrosis or hemorrhage was seen in 3 cases.

**Table 2:** FRS2 immunostaining and FISH results in 91ALT/WDLs and 91 DDLs

***FRS2*** FISH	FRS2 immunostaining				Total
	-(%)	1+ (%)	2+ (%)	3+ (%)	
*FRS2* amplified	6 (3.5%)	46 (26.7%)	63 (36.6%)	57 (33.2%)	172
ALT/WDL	3 (3.6%)	31 (36.9%)	31 (36.9%)	19 (22.6%)	84
DDL	3 (3.4%)	15 (17.0%)	32 (36.4%)	38 (43.2%)	88
*FRS2* non-amplified	5 (50%)	5 (50%)	0 (0%)	0 (0%)	10
ALT/WDL	5 (71.4%)	2 (28.6%)	0 (0%)	0 (0%)	7
DDL	0 (0%)	3 (100%)	0 (0%)	0 (0%)	3

*ALT/WDL* Atypical lipomatous tumor/well-differentiated liposarcoma, *DDL* Dedifferentiated liposarcoma, *FISH* fluorescence in situ hybridization, − negativity, *1+* weak positivity, *2+* moderate positivity, *3+* strong positivity

#### Immunostaining results in *FRS2*-nonamplified cases

Among 10 *FRS2* gene-nonamplified cases (7 ALT/WDLs and 3 DDLs), the IHC results of 5 cases were in agreement with FISH results, showing negative FRS2 immunostaining. However, 5 cases were FRS2-immunopositive, with 1+ positivity. The 5 FRS2-immunopositive cases were composed of 2 ALT/WDLs and 3 DDLs, among which 2 cases had necrotic areas and 1 sample had hemorrhagic areas.

All 64 control samples were *FRS2* nonamplified, while 17 of them (26.5%) were FRS2-immunopositive. Two cases had 2+ staining, including 1 low-grade myofibroblastic sarcoma and 1 pleomorphic liposarcoma, and the latter was polysomic for CEP12. In the 15 cases with 1+ staining, 2 tumors also harbored a polysomic pattern (1 pleomorphic liposarcoma and 1 undifferentiated pleomorphic sarcoma). Other changes such as necrosis or hemorrhage were found in 2 leiomyosarcomas, 1 pleomorphic liposarcoma, 1 lipoma, and 1 spindle cell lipoma.

### Diagnostic values of FRS2 immunostaining for ALT/WDL/DDL

The diagnostic sensitivity and specificity of FRS2 IHC in distinguishing ALT/WDL/DDL from other histological mimics were 94.0 and 73.5%, respectively (Table 3). With the standard of 2+ staining, the specificity reached 96.9%, while the sensitivity decreased to 66.0%. The sensitivity and specificity of FRS2 IHC for distinguishing ALT/WDL from normal fat tissue and other benign adipose tumors (lipoma, spindle cell lipoma) were 91.2 and 80.0%, and those of 2+ FRS2 were 54.9 and 100.0%, respectively. In the differentiation of DDL from other spindle cell tumors, the sensitivity and specificity were 96.7 and 65.5%, 76.9 and 93.1% by using 1+ staining and 2+ staining as cutoff values, respectively.

**Table 3:** Sensitivity and specificity of FRS2 immunostaining in diagnosing ALT/WDLs and DDLs

Tumor type	FRS2			
	≥1+		≥2+	
	Sensitivity	Specificity	Sensitivity	Specificity
ALT/WDL/DDL VS. control group	94.0%	73.5%	66.0%	96.9%
ALT/WDL VS. BAT & normal adipose tissue	91.2%	80.0%	54.9%	100%
DDL VS. other spindle cell tumors	96.7%	65.5%	76.9%	93.1%

*ALT/WDL* Atypical lipomatous tumor/well-differentiated liposarcoma, *DDL* Dedifferentiated liposarcoma, *BAT* benign adipocytic tumor, *1 +* weak positivity, *2+* moderate positivity;

### Comparison of FRS2 IHC results with classic diagnostic IHC markers in ALT/WDL/DDL and control cases

**Fig. 4:**
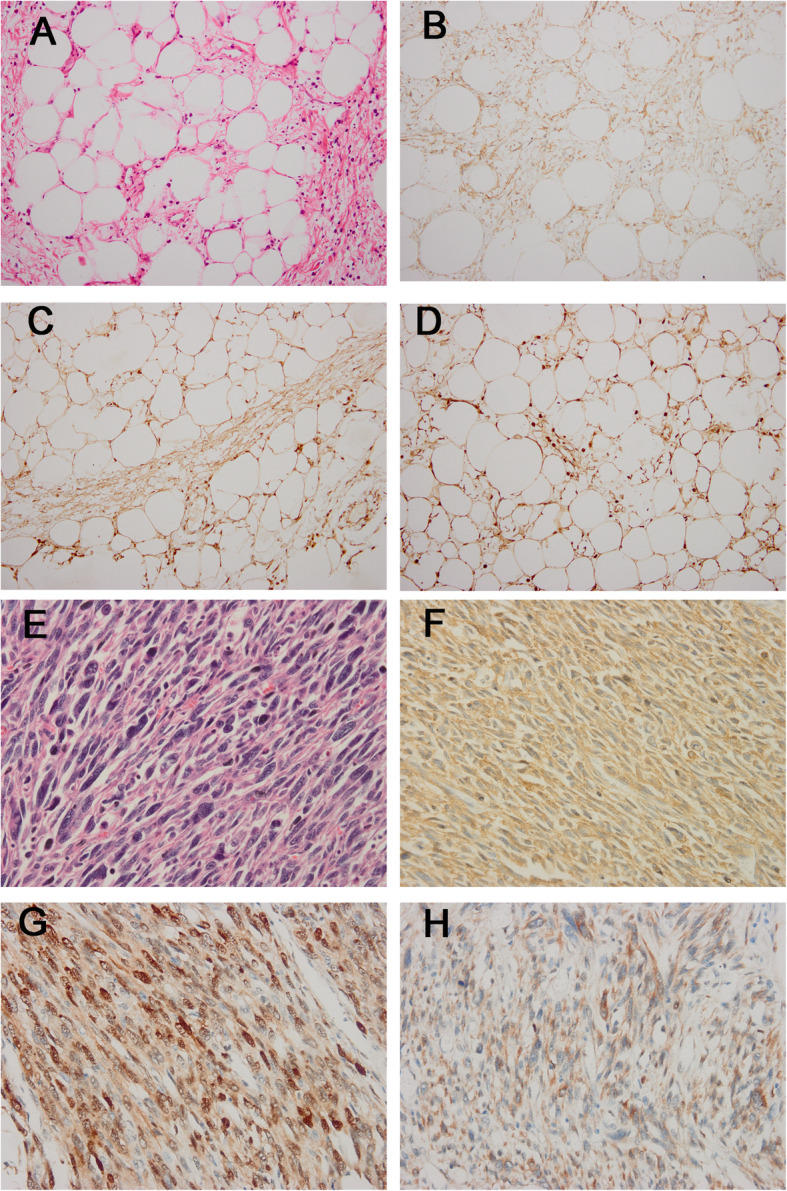
The histologic features of ALT/WDL and DDL and the corresponding FRS2, MDM2 and CDK4 immunohistochemical staining results. ALT/WDL (**a**) exhibited strong FRS2 immunostaining (**b**) and showed positive MDM2 (**c**) and CDK4 (**d**) immunostaining results. DDL(**e**) had strong FRS2 (**f**) immunostaining and positive MDM2 (**g**) and CDK4 (**h**) immunostaining results. (a, e, haematoxylin and eosin; b-d,f-h, immunostaining) (original magnification, a-d × 200, e-h × 400)

In our series, 177 tumors had prior MDM2 immunostaining results, and 164 had CDK4 results (Fig. 4). Among the 177 cases, all displayed MDM2 nuclear staining (100.0%), including 86 ALT/WDLs and 91 DDLs, among which 98 cases (55.4%) with 3+ positivity, 52 cases (29.4%) with 2+ positivity and 27 (15.2%) tumors displayed 1+ staining. The CDK4 immunostaining results showed that 159 of 164 cases (97.0%) were positive for CDK4, containing 77 ALT/WDLs and 82 DDLs, with 81 cases (49.4%) showing 3+ positivity, 43 cases (26.2%) with 2+ positivity and 35 cases (21.4%) with 1+ staining. In ALT/WDL/DDLs, the sensitivity of FRS2 immunostaining was generally similar to that of MDM2 (FRS2 vs. MDM2: 94.0% vs 100.0%) and CDK4 (FRS2 vs. CDK4: 94.0% vs 97.0%) and slightly lower than these two classic immunomarkers. Moreover, FRS2 immunostaining was positive in 166 of 172 *FRS2* gene-amplified cases, with a consistency rate of 96.5%, which was similar to the consistency rate of MDM2 immunostaining (100%).

MDM2 and CDK4 IHC was performed in 59 control cases with available material. The MDM2 immunostaining result showed 40 cases (67.8%) were negative for MDM2 and 19 were positive, including 16 displaying 1+ positivity and 3 with 2+ positivity. The CDK4 IHC result revealed 38 cases (64.4%) were negative for CDK4 and 21 cases were positive, among which 17 cases had 1+ positivity and 4 cases showed 2+ positivity. The specificities of MDM2 and CDK4 immunostaining were 67.8 and 64.4%, respectively, slightly lower to that of FRS2 immunostaining which was 73.5%.

## Discussion

The diagnosis of ALT/WDL/DDL based only on histological features could sometimes be challenging. Owing to the consistent amplification of *MDM2* and *CDK4* in ALT/WDL/DDL, FISH and IHC for the identification of *MDM2* and *CDK4* amplification or overexpression have been widely used for the differential diagnosis of this entity [7–13]. Recently, the amplification of the *FRS2* gene in ALT/WDL/DDL was identified by several studies, including our research group [14–16]. The use of FRS2 IHC was assessed by Zhang et al. in only 11 DDLs [15]. Here, we performed FRS2 IHC in 182 ALT/WDL/DDL and 64 histologic mimics with corresponding genetic data.

In the present study, 172 of 182 (94.5%) ALT/WDL/DDL showed *FRS2* amplification by FISH, and 10 cases were *FRS2-*nonamplified but *MDM2*-amplified. The high frequency of *FRS2* amplification of ALT/WDL/DDL in our research was similar to the results of 2 previous studies, with amplification frequencies of 100 and 93%, respectively [14, 15]. Immunostaining results showed that 171 of 182 (94.0%) ALT/WDL/DDL cases were positive for FRS2, including 83 ALT/WDLs and 88 DDLs. The positivity of FRS2 IHC (94.0%) was similar to the positivity of *FRS2* FISH (94.5%) in 182 cases, with relatively good consistency (*P* < 0.05, kappa = 0.444). In 172 *FRS2*-amplified cases, 166 (96.5%) cases were positive for FRS2 IHC, including 120 tumors (69.8%) demonstrating 2/3+ positivity. The high positive rate of FRS2 immunostaining in ALT/WDL/DDL indicated that FRS2 IHC could be a screening tool for ALT/WDL/DDL cases. It should be mentioned that 6 *FRS2*-amplified cases were FRS2-immunonegative, consisting of 3 ALT/WDLs and 3 DDLs. In the 6 FRS2-immunonegative cases, effect of storage time and tumor necrosis or hemorrhage of some samples (5/6) may be related to false negative results. In 10 *FRS2*-nonamplified cases, 5 cases were also FRS2-immunonegative, while 5 cases showed 1+ FRS2 positivity, including 2 ALT/WDLs and 3 DDLs. The necrotic area and hemorrhagic area were identified in 3 of the 5 FRS2-immunopositive cases, which might be associated with false positive results.

In 64 *FRS2-*nonamplified control cases, 47 (73.5%) cases were negative for FRS2 immunostaining and 17 were FRS2-immunopositive, including 2 cases with 2+ positivity and 15 cases with 1+ positivity. It should be mentioned that the FISH analysis of the pleomorphic liposarcoma with 2+ positivity showed polysomic for CEP12, which may lead to FRS2-immunopositivity in this case. Moreover, among the 15 cases with 1+ FRS2 expression, 2 tumors also harbored a polysomic pattern (1 pleomorphic liposarcoma and 1 undifferentiated pleomorphic sarcoma). Other changes such as necrosis or hemorrhage were found in 5 cases (2 leiomyosarcomas, 1 pleomorphic liposarcoma, 1 lipoma and 1 spindle cell lipoma). Therefore, the evaluation should be careful in the cases exhibiting 1+ positivity with hemorrhage and/or necrosis.

This series showed that the diagnostic sensitivity and specificity of FRS2 IHC were 94.0 and 73.5%, respectively, in distinguishing ALT/WDL/DDL from other histological mimics. The results suggested that FRS2 could be used as a screening tool for ALT/WDL/DDL. In addition, we analyzed the utility of FRS2 IHC for differentiating ALT/WDL and DDL from their own morphological simulators. When we used 1+ or 2+ as the threshold value, the sensitivity and specificity of FRS2 to distinguish ALT/WDL from fat tissue and benign adipocytic tumors were 91.2 and 80.0%, 54.9 and 100%, respectively. In differentiating DDL from other spindle cell tumors, the sensitivity and specificity of FRS2 were 96.7 and 65.5%, respectively. Interestingly, when using 2+ staining as the cutoff value in the differentiation of DDL with its histologic mimic, the sensitivity and specificity of FRS2 IHC changed to 76.9 and 93.1%, respectively. Hence, when one case had 2+ FRS2 immunostaining positivity, ALT/WDL/DDL should be highly suspected.

The total positive rate of FRS2 immunostaining in our cohort was generally similar to that of existing diagnostic markers (MDM2 and CDK4). In our series, the sensitivity of FRS2 immunostaining seemed to be slightly lower than that of MDM2 (FRS2 vs. MDM2: 94.0% vs 100.0%) and CDK4 (FRS2 vs. CDK4: 94.0% vs 97.0%), whereas the sensitivities of MDM2 and CDK4 in previous studies ranged from 45 to 100% and 41 to 100%, respectively [9, 11, 12, 21–23]. It should be emphasized that MDM2 and CDK4 also could show false positivity in the *MDM2*-nonamplified cases and the specificity of MDM2 and CDK4 immunostaining ranged from 60 to 100% in different studies [11, 12, 21, 23–28]. It should be noted that in this study the specificity of FRS2 immunostaining was slightly higher than the result of MDM2 (FRS2 vs. MDM2: 73.5% vs 67.8%) and CDK4 (FRS2 vs. CDK4: 73.5% vs 64.4%).Therefore, FRS2, MDM2 and CDK4 may be complementary in the diagnosis of ALT/WDL/DDL. Notably, molecular analysis should be considered for challenging cases.

The main therapeutic strategy for ALT/WDL/DDL is surgical resection; in the meantime, targeted therapy has been developed and is worthy of further exploration, especially for unresectable tumors. In recent years, MDM2 antagonists and CDK4 inhibitors have been used in clinical and preclinical studies, and some of them obtained favorable outcomes [29–32]. In our study, the analysis of *FRS2* gene amplification and expression can help in the diagnosis of ALT/WDL/DDL. Moreover, as the key adaptor of the FGFR pathway, the overexpression of FRS2 in ALT/WDL/DDL indicated that it could play an important role in the targeted therapy of this disease. Zhang et al. identified activation of FGFR/FRS2 pathways in liposarcoma, and the use of FGFR inhibitors could significantly inhibit the growth of liposarcoma cells. Other groups also reported that FGFR inhibitors could inhibit cell proliferation in *FRS2*-amplified DDL cell lines, further demonstrating the possible therapeutic use of the FGFR/FRS2 pathway in ALT/WDL/DDL [15, 33, 34]. In addition, recent studies showed that pharmacologically targeting FRS2 inhibited FGF/FGFR-mediated oncogenic signaling and tumor progression in prostate cancer and gastric cancer cell lines [35]. Further investigations are warranted to further evaluate the therapeutic prospects of the FGFR/FRS2 pathway in ALT/WDL/DDL.

In summary, our study analyzed the expression of FRS2 protein in 182 ALT/WDL/DDL cases and compared the result with genetic data. The FRS2 immunostaining had relatively good consistency with *FRS2* FISH. Moreover, FRS2 immunostaining had a slightly lower sensitivity but a slightly higher specificity than that of classic IHC markers (MDM2 and CDK4). It also should be noted that FRS2 IHC performed in a similar fashion to MDM2 and CDK4, which are also imperfect in their way with a subset of cases showing false immunopositivity in *MDM2*/*CDK4*/*FRS2*-nonamplified samples. This new marker could be utilized as an additional screening tool for the diagnosis of ALT/WDL/DDLs in laboratories without the possibility of performing in situ hybridization assays. Importantly, careful histological inspection, immunohistochemical and molecular tests can help to arrive at correct diagnosis for challenging cases. It must be emphasized that *MDM2/CDK4/FRS2* especially *MDM2* FISH remains the gold standard and the most recommended method to diagnose this entity. In the future, the amplification and overexpression of the *FRS2* gene in this disease spectrum might provide new clues for the targeted therapy of ALT/WDL/DDL.

## Data Availability

All data generated or analysed during this study are included in this published article and its supplementary information files.

## Abbreviations

FRS2: Fibroblast growth factor receptor substrate 2
ALT: Atypical lipomatous tumor
WDL: Well-differentiated liposarcoma
DDL: Dedifferentiated liposarcoma
FISH: Fluorescence in-situ hybridization
IHC: Immunohistochemistry
MDM2: Murine double minute 2
CDK4: Cyclin-dependent kinase 4
FNCLCC: Federation Nationale des Centres de Lutte Contre le Cancer
